# The effect of ammonia concentration on the treatment of bio electrochemical leachate using MFCs technology

**DOI:** 10.1007/s11356-023-31472-x

**Published:** 2023-12-28

**Authors:** Aliyu Ishaq, Mohd Ismid Mohd Said, Shamila Binti Azman, Mohd Firdaus Abdulwahab, Mohamad Rajab Houmsi, Zainab Toyin Jagun

**Affiliations:** 1 Department of Water Resources and Environmental Engineering, Ahmadu Bello University, Zaria, Nigeria; 2 Department of Biosciences, Faculty of Sciences, Universiti Teknologi Malaysia, Johor Bahru, Malaysia; 3 Centre for River and Coastal Engineering, 81310 Johor Bahru, Malaysia; 4 Department of Real Estate, School of Built Environment Engineering and Computing, Leeds Beckett University, City Campus, Leeds, UK

**Keywords:** Microbial fuel cell, Ammonia nitrogen, Inhibition, Electricity, Sustainability, Treatment

## Abstract

Microbial fuel cells (MFCs) have garnered attention in bio-electrochemical leachate treatment systems. The most common forms of inorganic ammonia nitrogen are ammonium (${NH}_{4}^{ +}$) and free ammonia. Anaerobic digestion can be inhibited in both direct (changes in environmental conditions, such as fluctuations in temperature or pH, can indirectly hinder microbial activity and the efficiency of the digestion process) and indirect (inadequate nutrient levels, or other conditions that indirectly compromise the microbial community’s ability to carry out anaerobic digestion effectively) ways by both kinds. The performance of a double-chamber MFC system—composed of an anodic chamber, a cathode chamber with fixed biofilm carriers (carbon felt material), and a Nafion 117 exchange membrane is examined in this work to determine the impact of ammonium nitrogen (${NH}_{4}-N$) inhibition. MFCs may hold up to 100 mL of fluid. Therefore, the bacteria involved were analysed using *16S rRNA*. At room temperature, with a concentration of 800 mg L^−1^ of ammonium nitrogen and 13,225 mg L^−1^ of chemical oxygen demand (COD), the study produced a considerable power density of 234 mWm^−3^. It was found that ${NH}_{4}-N$ concentrations above 800 mg L^−1^ have an inhibitory influence on power output and treatment effectiveness. Multiple routes removed the most nitrogen (${NH}_{4}^{ +}$*-N: 87.11* ± *0.7%, NO*_*2*_^*–*^*N: 93.17* ± *0.2% and TN: 75.24* ± *0.3%).* Results from sequencing indicate that the anode is home to a rich microbial community, with anammox (6%), denitrifying (6.4%), and electrogenic bacteria (18.2%) making up the bulk of the population. Microbial fuel cells can efficiently and cost-effectively execute anammox, a green nitrogen removal process, in landfill leachate.

## Introduction

The disposal of municipal solid waste results in leachate from landfills. It is made up of various nutrients, chemicals, pollutants, and metals, both organic and inorganic. Leachate from landfills contains various contaminants, including organic compounds, ${NH}_{3}-N$, heavy metals, chlorinated organic and inorganic compounds, salts, and other pollutants. Potential surface and groundwater contamination are from leachate’s heaviest contaminants. Large quantities of leachate are produced as a byproduct of the common landfill practice in Malaysia to dispose of solid waste. Leachate from organic waste can increase soil fertility, but it also smells bad, attracts pests, and causes off-gassing (Wijekoon et al. 2022). Pollutant content and concentration in leachate are largely affected by the landfill’s age (Tatsi and Zouboulis 2002). Its characteristics change because of its age, precipitation, seasonal weather variance, waste type, and composition. In contrast to mature landfill leachates, which typically contain a high concentration of ammonium and non-biodegradable organic matter (dominated by humic and fulvic acids) (Renou et al. 2008), young landfill leachates with high COD values and a *BOD*_*5*_*/COD* ratio of 0.4 are more than mature.

Leachate from landfills may be cleaned up using a variety of treatment methods. Various techniques, such as anaerobic and aerobic digestion, ultrafiltration, nanofiltration, reverse osmosis, coagulation-flocculation, chemical precipitation, and ammonia stripping (Kurniawan et al. 2006), can be used to treat leachate. Although a biological treatment strategy would be ideal due to leachate’s high biodegradability, its treatment efficiency is severely limited by substantial concentrations of ammonia, sulphides, and metals (Chang et al. 2018). There is evidence in the literature that certain oxidants (*O*_*3*_, *H*_*2*_*O*_*2*_, *S*_*2*_*O*_*8*_) catalysts (*TiO*_*2*_, *ZnO* ${Fe}^{2+}$) and their coupling are used in advanced oxidation processes (AOPs) (Hassan et al. 2018). Unfortunately, landfill leachate’s complexity means they are not being used to their full potential. Most recently, studies (Hassan et al. 2018) successfully removed refractory organics and ammonia from aged landfill leachate using a combination of bioreactor-based biological treatment and photo-irradiated titanium dioxide and persulphate oxidation-based pos post-treatment, although these cutting-edge treatment strategies are expensive and challenging to several technological limitations that remain, such as the need to separate the catalyst further once the water has been treated and the depth to which light may penetrate an aqueous photocatalyst suspension.

To meet discharge quality criteria while reducing the volume of leachate effluent, it is necessary to pick certain simple and inexpensive treatment processes, especially in developing countries like Africa, where open trash dumping attracts rain and produces leachate wastewater (Ishaq et al. 2023). One of the most promising electrochemical wastewater treatment technologies, microbial fuel cells (MFC), have the potential also to supply humanity with clean energy. Wastewater contains organic compounds that microorganisms can convert into energy in MFC (Zhao et al. 2023). Safe, clean, efficient, and direct energy generation are just two of the many advantages of MFC for wastewater treatment and eliminating organic pollutants. A proton exchange membrane (PEM) often separates the anode and cathode chambers in MFCs. Oxidation of the substrate by bacteria results in the release of protons and electrons. Protons are transferred using a PEM, while electrons use an external circuit.

Several studies have shown that MFCs may be used to efficiently treat landfill leachate by removing contaminants such as ammonia nitrogen ${(NH}_{4}^{+}$*),* total nitrogen, and chemical oxygen demand (COD) (Hassan et al. 2018; Zhang et al. 2015). Power densities reported for MFCs generating electricity from landfill leachate range from 5.5 to 824 mW/m^3^ (Hussein et al. 2021; Jiang et al. 2019; Yaashikaa et al. 2022). These values vary widely based on a wide variety of factors, including substrate concentration, reactor type, pH, inoculum concentration, the nature of micro-ammonia (${NH}_{3}$) acts as a proton acceptor, decreasing the availability of protons in the anode compartment, and slows current generation by interfering with electron transfer mechanisms inside microbial populations (Deng et al. 2021). It was previously believed that denitrification was the principal biological mechanism responsible for the conversion of reactive inorganic nitrogen species to *N2*. In the anammox process, ammonium and nitrite are reacted directly to produce nitrogen gas *N2 (*${NH}_{4}^{+}$*-N, *${NO}_{2}^{-}$_–_*N → N*_*2*_) bypassing the need for oxygen or organic material (Kartal et al. 2013).

Nitrification occurs when an abundance of oxygen converts ammonium to nitrate (Virdis et al. 2011; Zhang and He 2013). In this method, ammonium is directly reduced to nitrogen gas via denitrification after being oxidised to nitrite without first oxidising to nitrate (W. W. Li et al. 2014). Furthermore, even when aerobic conditions are present, nitrification and denitrification can occur concurrently in a single reactor (Virdis et al. 2011). The processes of nitrification and denitrification occur at the same time. The primary objectives are to research the viability of employing anaerobic ammonia oxidation (anammox) for biofilm growth in landfill leachate treatment and determine the optimal ammonia nitrogen concentration that inhibits MFC function. In this understudied area, more investigation into the inhibitory mechanism, the identification of electron-producing bacteria, and their role in therapeutic efficacy.

The optimal concentration of ammonia nitrogen that inhibits MFC performance is the subject of ongoing studies. This method developed biofilm growth with anaerobic ammonia oxidation (anammox). Little was made of this discovery. Thus, the inhibitory mechanism needs to be studied. The anode chamber substrate in this work used landfill leachate. The feasibility of removing organic/ammonia nitrogen while producing power was investigated. The anodic chamber’s microbiome was studied and identified as well as impact nitrogen removal and power generation.

## Materials and test methods

### Sampling

In a 50-L clean plastic container, fresh samples of leachate wastewater and sludge were manually collected at the Simpang Renggam Landfill Site (SRLs), Johor, Malaysia. After collection, the samples were delivered to the lab and cooled to 40 °C before the first characterisation. The pH and the temperature were measured where the samples were collected. Only top-notch analytical reagents were used in this analysis. Within 48 h, APHA (2005) was used to quantify and describe the leachate’s temperature, biochemical oxygen demand (BOD), chemical oxygen demand (COD), ammoniacal nitrogen (${NH}_{3}-N$), total dissolved solids (TDS), nitrite, nitrate, alkalinity, *BOD*_*5*_*/COD*, and microbiological analysis. Table 1 lists the characteristics of the leachate.

**Table 1:** Characteristics of landfill leachate used in this study

Parameter	Unit	Concentration
BOD	mg L^−1^	112 ± 7
COD	mg L^−1^	1322.5 ± 5
BOD_5_/COD	-	0.0845 ± 1.4
NH_4_-N	mg L^−1^	411 ± 6
Turbidity	NTU	134.95 ± 8
pH	-	6.88 ± 3
Temperature	^0^c	30 ± 2.5
Conductivity	µs.cm	107,746 ± 7.4
NO_3_-N	mg L^−1^	70 ± 5.5
NO_2_-N	mg L^−1^	110 ± 8.5
Salinity	ppt	5.58

### MFC configurations and operation mode

With a few minor adjustments (reactor adjustment scale), MFCs (H-type dual-chambered sets) with specifications comparable to those in other research (Nor et al. 2015; Hassan et al. 2018) were used in the experimental phase. The transparent Plexiglas MFCs were constructed by the schematic layouts of the laboratory-scale reactors utilised in this investigation. Each MFC had a cathode compartment and an anode compartment, sized 5 cm × 5 cm × 4 cm, with a working volume of 100 mL. The anode and cathode electrodes were made of carbon felt, which have the following measurements: *h* = 1.7 cm, *w* = 0.6 cm, and *l* = 1.7 cm. The proton exchange membrane (PEM) Nafion 117 divided the electrodes, spaced apart by about 4 cm.

### Pre-treatment of Nafion membrane

Pre-treatment of the Nafion 117 membrane (DuPont, DE, USA) involved soaking it in 0.1 M H_2_ S0_4_, then 0.1 MH_2_ 0_2_, and rinsing it with deionised water. Each procedure took 60 min at 60 °C (Bakhshian et al. 2011).

#### Pre-treatment of anode, cathode materials

The MFCs setup was submerged in 5% ethanol (*C*_*6*_*H*_*6*_*O* for 10 min) to prevent contamination. They were then completely rinsed with distilled water and exposed to UV light for 15 min (Nor et al. 2015). To enhance porosity and lessen the impact of impurities, the anode and cathode were completely submerged in acetone for 24 h, cleaned with deionised water, and then dried in the open air overnight (Özkaya et al. 2013).

#### Setting up the MFCs

**Fig. 1:**
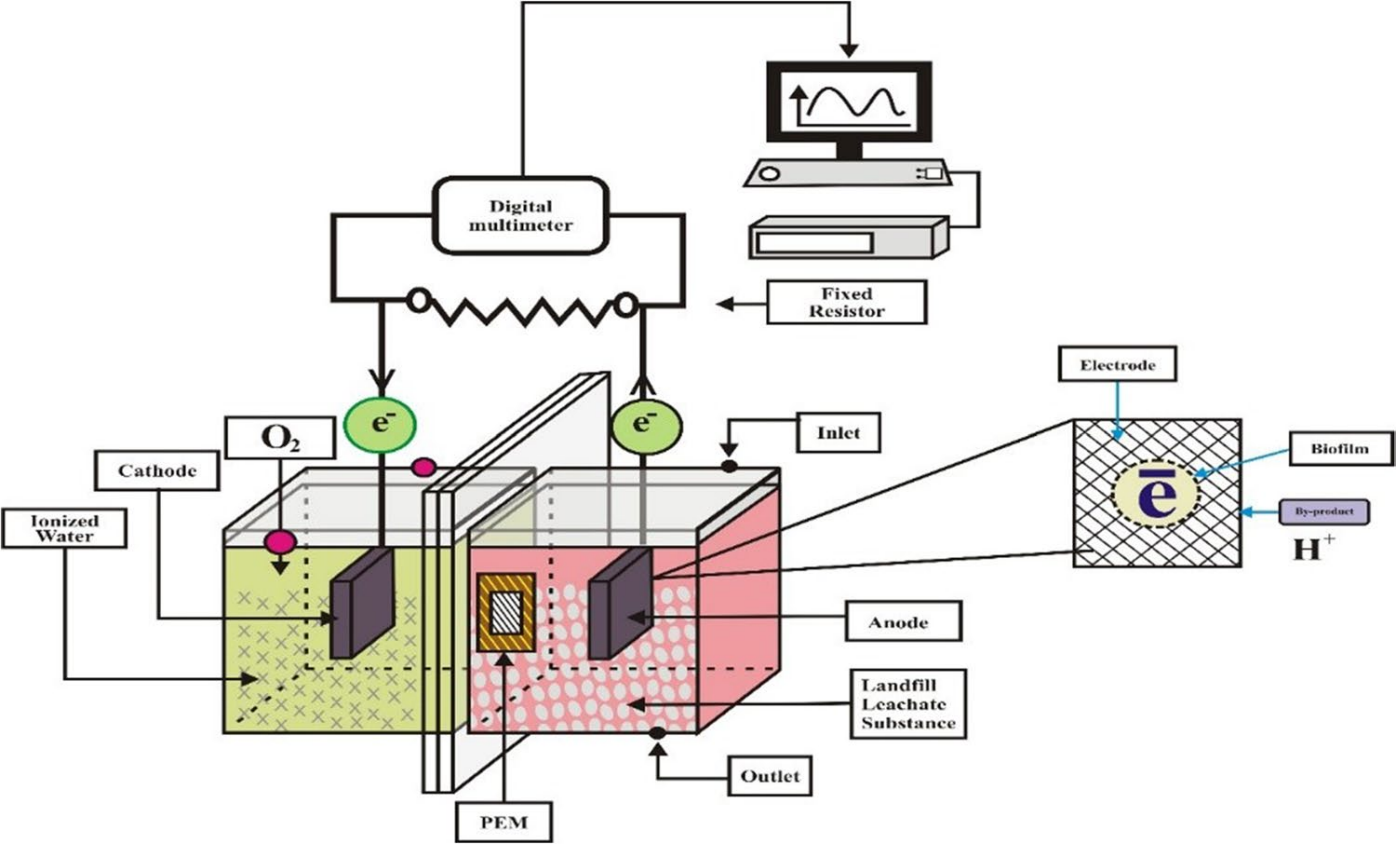
Schematic diagram of double chamber MFC

MFCs were coupled with gum and nuts to stop the leakage (see Fig. 1). According to Coppath Hamza et al. (2017), all reactors have their anode sides closed and their cathode sides left open. Sampling ports were made on the side of the reactor so samples could be quickly collected and examined. The electrodes were connected in series with a copper wire as a current collector. Both electrodes were cleaned with ethyl alcohol before being rinsed with distilled water to remove impurities. The anode chamber was sparged with nitrogen gas to ensure anaerobic conditions and oxygen removal during the MFC’s beginning (Jagaba et al. 2021). Leachate served as the feeding substrate in each anolyte chamber. The electrodes were attached to a copper wire as a current collector. A closed electrical circuit was created by attaching electrodes to a resistor box with an external resistance set at 1 k Ω. Every 30 s, the generated voltage was measured using a digital multimeter (LABJACK U3-HV) linked to a computer. During the experiment, distilled water was used to fill the catholyte chamber, and constant aeration was achieved at the top of the cathode chamber with an aquarium pump to provide mixing and feed air bubbling for 24 h.

### Biofilm development for electroactive bacteria

The substrate was changed occasionally (in circles) to help with acclimatisation. The cycles were repeated until the voltage maximum was reached across 1000 resistors. Leachate was used for the studies in this investigation exactly as it was collected, without any pH modifications, nutritional or trace metal additions, or dilution. To enrich the MFCs with ammonium-oxidising bacteria (AOB), *(*${NH}_{4}$*)*_*2*_*SO*_*4*_ and ${NaNO}_{2}$ were added to the leachate since it was old to maintain the ratio of ${NH}_{4}^{+}$*-N* and ${N0}_{2}^{-}$*-N* ratio at 1:1.32 (Hassan et al. 2018). To enrich the bacterial consortia that served as a source of inoculation due to the presence of the appropriate mixed bacterial community, 10% v/v of sludge collected from the anaerobic secondary digester of Simpang Renggam Wastewater Treatment Plant in Johor, Malaysia, was added to the respective MFCs (Damiano et al. 2014). The substrate in MFCs was continuously altered at the same percentage during acclimatisation to enrich the system with anammox (ammonium-oxidising bacteria). A peristaltic pump was used to provide the anode chamber with leachate wastewater constantly, and the hydraulic retention time (HRT) was adjusted to 4 h. This HRT was used to increase reactor start-up speed and to enhance biofilm development (Ichihashi et al. 2014). The experiment was conducted in a room with a temperature range of 27–30 °C.

### MFC operation, inoculation, and batch operation mode

The experiment was divided into three independent phases or components. The MFCs system was operated for 38 days to enrich microorganisms during the first phase of the research and for 21 days at an open circuit voltage (no findings were presented). The second step, which begins after the acclimatisation period, entails utilising the OFAT technique to feed various NH_3_ influent fractions at different concentrations for 48 days. This was accomplished to track the system’s power output, treatment effectiveness, and anaerobic ammonium oxidative denitrification capacities. Multiple runs of each experiment were performed. The cathode’s electron acceptor was filled with pure water throughout the experiment. On the other hand, the air was used as the supply and deionised water as the catholyte throughout the research. The study could focus solely on the anode’s performance as a result. The generation of the internal current is facilitated by the occurrence of electrochemical reactions at both the anode and cathode electrodes. The presence of deionised water in the cathode chamber electrolyte does not inherently engender the production of current. Nonetheless, the deionised water functions as a medium through which the process of oxygen reduction reactions occurs at the cathode. This procedure involves the oxidation of organic substance at the anode and the consequent decrease of oxygen at the cathode. The completion of the circuit occurs when the electrons, having travelled through the external circuit, reach the cathode and participate in the reduction of oxygen.

The number of COD in each MFC—starting with the control—was monitored throughout the study’s duration. The pH level was measured and adjusted once judged within the permissible range. The MFC was operated without internal pH regulation at room temperature, which varied from 27 to 30 °C. To compare the impact of the various MFCs, one MFC is utilised as a control (without inoculation). 1, 2, 3, and 4 cycles later. The effluent’s water quality was assessed, and the results for each parameter’s average value were given. The usual atmospheric pressure was used for the experiments, which were conducted at temperatures between 27 and 30 °C.

### Operating procedures with monothetic analysis method

The monothetic analysis, also known as one-factor-at-a-time (OFAT method), is a technique for designing experiments that simultaneously test one element, or cause, at a time rather than many factors. With different ${NH}_{3}$ infraction concentrations, this work investigated monothetic analysis for double-chamber MFCs. To find out how ammonia affects electricity generation in MFCs between 400 and 1600 mg L^−1^ while maintaining a constant COD concentration of 1322.5 and pH of 6.88 at room temperature. The values for total nitrogen (TN), ammonium nitrogen ${(NH}_{4}^{+}$*-N*) and nitrite $({NO}_{2}^{-}$*−N*), and nitrate-nitrogen (${NO}_{3}^{-}$*−N*) were calculated using the APHA standard methods (2005). Each experiment was conducted in duplicate.

### Leachate characterisation, analytical determinations, and computation

Total nitrogen, ammonium, chemical oxygen demand, pH, nitrite, and nitrate concentrations were measured to assess treatment efficacy. Each one was examined using criteria established by the American Public Health Association (2005). To cut down on mistakes and boost precision, we ran all analyses twice and averaged the results for each parameter.

#### Electricity performance

The steady voltage potential was recorded after 60 s with no external resistance (open circuit voltage). To record voltage, current, and power, 1k resistors were connected to a lab jack U3-V Multimeter (Agilent Technologies, CA, USA) with data logging capability. The power density was determined by plugging $\left(P=IV\right)$ into the formula $P=\frac{p}{A}$, while the current was determined by plugging in $I=\frac{V}{R}$, where electrode surface area is *A*, voltage is *V*, current is A/m^2^, and power is *w*. The formula for calculating current density $j=\frac{I}{A}$ where *J* is the current density; *I* is the current (A), and *A* is the electrode surface area (m^2^). A steady state has been reached when this parameter maintains a steady value.

#### Polarisation curve

The voltage of an MFC system is represented by a curve called the polarisation curve. This was determined by leaving the MFC electrode disconnected (open circuit) for 2 h and adjusting the external resistance of the circuit, starting with 5000 Ω, 3950 Ω, 3690 Ω, 3240 Ω, 2050 Ω, 1530 Ω, 1300 Ω, 900 Ω, 620 Ω, 580 Ω, 550 Ω, 500 Ω, 400 Ω, 390 Ω, 290 Ω, 250 Ω, 240 Ω, 190 Ω, 150 Ω,140 Ω, 130 Ω, and 100 Ω through a multimeter recorder (LABJACK U3-HV) that was hooked up to a computer for no longer than 20 min for each resistance and to monitor each drop in voltage as current increases (Nor et al. 2015). This method was repeated for all the MFCs concurrently under continuous fed-batch operation mode. The polarisation curve was determined by calculating the following:

The current, *I* (A), produced by the MFC was calculated according to Ohm’s law, where *V* (v) is the measured voltage, and *R* (Ω) is the external resistance:

1 $$I=\frac{V}{R}$$

The power output *P* (W) was calculated according to Eq. (2):

2 $$P=IV$$

*I* is the current determined by Eq. (1), and *V* is the voltage measured across the resistor load. According to Eq. (3), where *P* is the computed power (Eq. (2)), and *A* (m^2^) is the effective surface area of carbon cloth utilised as the anode, we obtain the power density PA (mWm^−2^)

3 $$P= \frac{P}{A}$$

The recovery of electrons is the Coulombic efficiency, CE (%), which was calculated using the Logan equation (Eq. (4)):

4 $${C}_{E}=\frac{8{\int }_{0}^{t}Idt}{F\Delta CODVAn}\times 100\%$$

where ΔCOD (COD_in_ − COD_out_) (mg L^−1^) is determined by deducting the COD concentration in the influent from that in the effluent, *F* (96,485 C/mol) is Faraday’s constant, *I* (mA) is the current across the resistor in the MFC reactor during operation time (*t*, *d*), and *V*_An_ is the volume of the working liquid in the anode chamber. The removal efficiencies of ammonia nitrogen, nitrite, nitrate, and total nitrogen for each batch cycle were determined according to Hassan et al. (2018).

### Microbial analysis

At the completion of the experiment, samples of the anode carbon felt were collected from two distinct places (E1 and E2) and subjected to high-quantity sequencing to identify the microbial population adhering to the anode. After each experiment, a sterile pipette tip was utilised to scrape the entire biofilm biomass off the anodes, and the collected sample of biofilm biomass was subsequently suspended in a sterile Scott bottle. Nanodrop spectrophotometer (ND 1000, Thermo Scientific) absorbance readings at 260 and 280 nm were used to evaluate the purity and concentration of the isolated DNA (Thermo Fisher Scientific, Beverly, MA, USA). The DNA was extracted from a fraction of the biomass (250 mg) using the DNeasy(R) Powersoil(R) Pro kit DNA extraction kit following the manufacturer’s instructions. Three parallel 25 L PCR reactions were performed, and the PCR results were pooled for electrophoresis purification. The details of the PCR procedure and sample preparation were described in the following (Li et al. 2014). The variety of microorganism communities was illustrated by examining their alpha diversity in a single sample. The abundance and diversity of bacterial species were evaluated using many statistical analysis indices calculated with the MOTHUR platform; they included the ACE index, Chao 1 index, Shannon index, Simpson index, and coverage. Low-quality and erroneous sequences in the dataset were validated and removed using the Mothur software (http://www.mothur.org). The remaining sequences were then assigned to OTUs with a classification accuracy of 97%.

## Results and discussion

Table 1 provides data comprises crucial water quality parameters for assessing environmental conditions. Notably, the concentrations of *BOD* (112 ± 7 mg L^−1^) and *COD* (1322.5 ± 5 mg L^−1^) indicate the organic and chemical pollutant loads in the water, with a low *BOD*_*5*_*/COD* ratio (0.0845 ± 1.4) suggesting limited biodegradability of organic matter. Ammonium nitrogen (${NH}_{4}-N$) is present at 411 ± 6 mg L^−1^, while turbidity stands at 134.95 ± 8 NTU, indicating the presence of suspended particles. High turbidity can impair light penetration, affecting photosynthesis and disrupting the habitat for aquatic life. The source of these particles should be further investigated to assess potential pollution sources. The pH level is slightly acidic at 6.88 ± 0.3, while the high conductivity (107,746 ± 7.4 µs/cm) suggests a notable presence of dissolved ions. High conductivity values may indicate an elevated concentration of salts or other ionic compounds, potentially stemming from industrial discharges or geological factors. Furthermore, nitrate nitrogen (${NO}_{3}-N$) is at 70 ± 5.5 mg L^−1^, and nitrite nitrogen (${NO}_{2}-N$) measures 110 ± 8.5 mg L^−1^, both contributing to the assessment of inorganic nitrogen levels. These nitrogen species can originate from agricultural runoff, industrial processes, or wastewater discharge, and their presence can lead to nutrient pollution, impacting water quality and aquatic ecosystems. The temperature of 30 ± 2.5 °C plays a vital role in aquatic ecosystems, affecting the metabolism of aquatic organisms, the solubility of gases, and biological processes. Monitoring temperature helps assess the overall health of the ecosystem and can provide insights into potential thermal pollution. MFCs possess the ability to withstand a certain range of salt concentrations, and the optimal conditions can differ depending on the microbial community and the intended use of the MFC. Even though the salinity levels (5.58 ppt) in this research are within the acceptable range for MFCs, it is important to note that the inhibitory effect of salt in landfill leachate and the inhibitory effect of ammonia on electrically active microorganisms are separate yet connected issues in the field of microbial activity, especially in processes like MFCs for wastewater treatment (Gokgoz et al. 2023). Elevated levels of sodium in landfill leachate can indeed hinder the growth and activity of microorganisms. Elevated salinity levels might potentially disrupt the integrity of microbial cell membranes, influence enzyme functionality, and induce osmotic stress, ultimately hindering the growth and activity of specific microorganisms. In the framework of MFCs, where microbial activity generates electrical current, the impedance of electrically active microorganisms as a result of high salt concentrations can significantly impact the performance of the MFC (Kumar et al. 2020). The inhibitory effects of salt and ammonia on microorganisms may interact, potentially leading to a cumulative adverse effect on microbial activity. Higher concentrations of salt can worsen the strain on microorganisms, making them more vulnerable to the inhibitory impacts of other substances, like ${NH}_{3}$. The comprehensive analysis of these water quality parameters provides critical information for understanding the condition of the water source, identifying potential pollution sources, and guiding environmental management and remediation efforts for this study.

### Power generation of landfill leachate in double-chambered microbial fuel cells (MFCs)

The leachate has the necessary microorganisms, and preliminary studies conducted by MFCs showed that energy might be generated from it. Optimal electrical output and treatment efficiency settings can only be determined by operating in a continuous feed batch mode (Rahimnejad et al. 2015). The polarisation plots and maximum power generation were recorded to assess the MFC reactor’s performance. In addition, the power density was normalised according to the anode’s surface area.

**Fig. 2:**
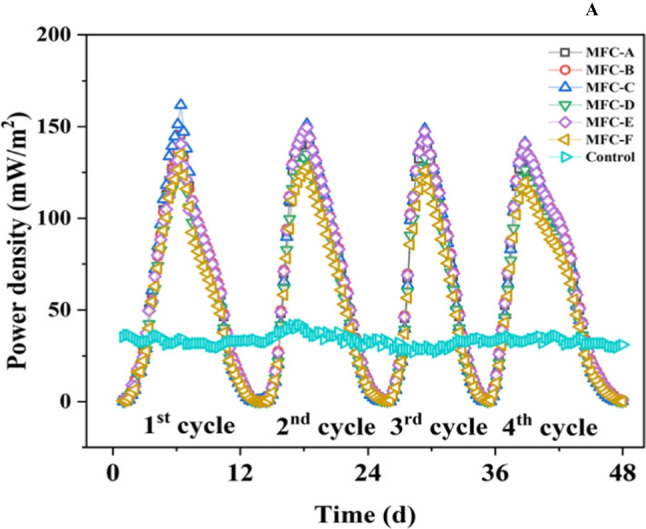
The power output evolutions plots against time (days) in the MFCs treating old landfill leachate for 48 days of experimentation in batch mode operation (phase 1) (Rext 1k Ω)

The MFCs were monitored through open circuit voltage (OCV) over time during the initial start-up, which helped to assess the stability and efficiency of the biofilm development and enrichment process, providing valuable information about the performance of the MFCs and the interactions between the microorganisms and the substrate. Each MFC observed an enormous decrease in voltage potential as the inoculation cells struggled to acclimatise to the new surroundings and increased immediately (data not shown). As the bacteria adapted, the cells recovered immediately. Figure 2 presents the results of the all the MFCs (MFC-A, MFC-B, MFC-C, MFC-D, MFC-E, MFC-F, and MFC-control), with maximum power densities of 161.81 mW ${{\text{m}}}^{-2}$, 149.89 mW ${{\text{m}}}^{-2}$, 149.64 mW ${{\text{m}}}^{-2}$, 145.55 mW ${{\text{m}}}^{-2}$, 135.01 mW ${{\text{m}}}^{-2}$, 134.99 mW ${{\text{m}}}^{-2}$, and 41.89 mW ${{\text{m}}}^{-2}$, respectively. The maximum power density across different MFCs is 161.81 mWm^−2^, while the control has a significantly low power density of 41.89 mWm^−2^. During the initial days, MFCs showed poor electricity generation, which could be the reason for the microorganism to get acclimatised in the system to form the biofilm. Once the microorganism gets acclimatised, the voltage starts to increase. In addition, the voltage drop trend would be due to the reduced availability of nutrients explained by Coppath Hamza et al. (2017). It can be observed that the power densities of the inoculated MFCs (A-F) are significantly higher than the control group, due to the absence of an active microbial community capable of generating electron flow. Among the inoculated MFCs, MFC A achieved the highest power density (161.81 mWm^−2^), suggesting that the specific biofilm development in MFC A was more effective in generating power than the other MFCs. This could be attributed to results variations since all the MFCs contained the same influent concentrations and inoculations except for MFC without inoculum. This agrees with the findings of Feng et al. (2020). Furthermore, several variables contribute to the differences in power densities observed for the seven MFCs using the same influent substrate conditions. The composition of initial microbial community within each MFC can differ since it is a mixed culture, there may have been abrupt changes in environmental conditions for some of the MFCs, there may have been potential contaminants or inhibitors in the substrates of some of the MFCs, hydrodynamic differences affecting mass transport, and there may have been different maintenance practices with the same substrate concentration (Li et al. 2020). This could also be as a result of presence or absence of certain species which may affect the biofilm formation, as well as its overall structure and function (Rahmani et al. 2022). The achievement of its highest power density of (161.81 mWm^−2^) could be the optimal balance between voltage output and current density, resulting in maximum power generation. At this point, the system is limited by mass transport, and the concentration gradients and diffusion of reactants and products constrain the performance. The polarisation curve provides insights into the power generation capabilities of the biofilm and the various losses that occur during the process (Logan 2010).

This variation can result in different colonisation patterns and, consequently, differences in biofilm development; differences in mixing and mass transfer rates between MFCs could affect the supply of nutrients, oxygen, and electron acceptors to the biofilm (Li et al. 2019). These differences can influence biofilm growth and activity, leading to variations in biofilm development between MFCs. Acclimatisation of the electrochemically active microorganisms growing on the anode surface is credited with the performance of continuous MFC systems because these bacteria must be viable at all phases of growth and must operate as the dominating population.

The table compares the effectiveness of the MFCs labelled A, B, C, D, E, and F and the control throughout several cycles and time intervals, considering factors such as operating time, Coulombic efficiency (CE), influent chemical oxygen demand (COD) concentration, percent COD removal, influent ammonium nitrogen (${NH}_{4}-N$*)* concentration, percent ${NH}_{4}-N$ removal, and total nitrogen (TN) removal percentage. The MFCs traversed cycles lasting 48 days, broken up into four groups of 7 days for biofilm growth at each cycle. All the MFCs are run under identical circumstances, except for the control group, which has not been inoculated. The percentage of COD and ${NH}_{4}-N$ removed by the MFCs increases as the number of cycles increases (see Table 2). This could be due to electrogenic bacteria populations in the MFCs growing and diversifying over time. The anode becomes colonised with more bacteria as MFCs run longer, enriching the anammox bacteria and increasing the number of microorganisms capable of oxidising organic materials. The % of COD reduction ranging from 23.9 to 55.7% with CE of 12.46–19.57% during the first two batches of each MFC. The removal efficacy of COD was primarily influenced by the power output. As explained in Table 2, COD removal ranged from 23.9 to 55.8% during the first two batches of each MFC, with the highest CE ranging from 12.46 to 19.76% when 90% leachate with 10% v/v inoculum were used. MFC C had the highest COD removal efficiency of 80.10 $\pm$ 0.5% (CE 12.04%) at operating cycle 3 during acclimatisation at 90% leachate substrate with 10% v/v inoculation, followed by MFC F with COD removal efficiency of 71.71 $\pm$ 0.5% (CE 9.69%). Electron-transfer bacteria, because to their low Coulombic efficiency, likely cannot convert all readily accessible carbon fuel into electricity (Hassan et al. 2018). The abrupt decline of CE between cycles 3 and 4 in all MFCs indicates the loss of substrate in a nonelectrical production process and that COD removal occurred predominantly in a non-electrogenic manner (Hassan et al. 2018). In MFC, however, methanogenic microbes compete with electrogenic microorganisms and convert the substrate to methane, thereby decreasing electron oxidation (Oliveira et al. 2013). Cecconet et al. (2018) reported that methanogenesis affects the Coulombic efficiency of MFCs, even though it may not directly impact organic removal. In contrast, the control (without inoculation) showed COD removal (22.44 $\pm$ 0.3–38.36 $\pm$ 0.0%), due to insufficient microbial activity in the anode chamber.

**Table 2:** Operating conditions and system performance for biofilm development at different cycles

MFCs	Cycles	Operating time (*d*)	CE (%)	Influents COD conc. (mg L^−1^)	% removal	Influent NH_4_-N conc. (mg L^−1^)	% removal	TN removal (%)
A	1	0–7	19.08	1327 ± 7.0	26.76 ± 0.5	411 ± 8.5	23.90 ± 0.1	18.21
	2	7–24	15.13	1327 ± 9.5	35.15 ± 0.3	411 ± 7.5	31.68 ± 0.2	28.31
	3	24–36	11.64	1327 ± 8.5	43.74 ± 0.5	411 ± 5.2	39.14 ± 0.1	31.23
	4	36–48	14.33	1327 ± 5.3	47.09 ± 0.5	411 ± 6.5	41.70 ± 0.0	20.54
B	1	0–7	19.76	1327 ± 7.0	32.43 ± 0.3	411 ± 8.5	32.14 ± 0.0	25.01
	2	7–24	13.74	1327 ± 9.5	47.80 ± 0.3	411 ± 7.5	44.85 ± 0.3	35.35
	3	24–36	11.52	1327 ± 8.5	55.64 ± 0.4	411 ± 5.2	51.82 ± 0.1	37.63
	4	36–48	13.88	1327 ± 5.3	59.92 ± 0.5	411 ± 6.5	55.31 ± 0.2	28.71
C	1	0–7	16.09	1327 ± 7.0	62.42 ± 0.3	411 ± 8.5	57.09 ± 0.2	34.41
	2	7–24	13.05	1327 ± 9.5	75.37 ± 0.5	411 ± 7.5	62.79 ± 0.2	45.05
	3	24–36	9.82	1327 ± 8.5	79.99 ± 0.3	411 ± 5.2	64.01 ± 0.1	51.61
	4	36–48	12.04	1327 ± 5.3	80.10 ± 0.5	411 ± 6.5	68.12 ± 0.0	58.76
D	1	0–7	15.55	1327 ± 7.0	52.81 ± 0.4	411 ± 8.5	45.87 ± 0.2	31.09
	2	7–24	12.72	1327 ± 9.5	61.76 ± 0.4	411 ± 7.5	55.33 ± 0.1	17.23
	3	24–36	9.93	1327 ± 8.5	69.29 ± 0.5	411 ± 5.2	58.79 ± 0.1	49.64
	4	36–48	12.31	1327 ± 5.3	69.85 ± 0.4	411 ± 6.5	61.50 ± 0.0	44.45
E	1	0–7	17.14	1327 ± 7.0	51.59 ± 0.5	411 ± 8.5	49.85 ± 0.1	30.12
	2	7–24	13.71	1327 ± 9.5	63.46 ± 0.3	411 ± 7.5	53.87 ± 0.2	50.25
	3	24–36	11.10	1327 ± 8.5	68.41 ± 0.9	411 ± 5.2	59.28 ± 0.1	37.98
	4	36–48	13.56	1327 ± 5.3	71.20 ± 0.2	411 ± 6.5	62.47 ± 0.0	41.43
F	1	0–7	15.73	1327 ± 7.0	57.56 ± 0.3	411 ± 8.5	50.36 ± 0.1	29.12
	2	7–24	12.46	1327 ± 9.5	69.91 ± 0.5	411 ± 7.5	55.82 ± 0.1	38.34
	3	24–36	9.69	1327 ± 8.5	71.71 ± 0.5	411 ± 5.2	60.36 ± 0.2	47.91
	4	36–48	12.05	1327 ± 5.3	70.20 ± 0.2	411 ± 6.5	64.55 ± 0.3	49.48
Contro l	1	0–7	18.81	1327 ± 7.0	22.44 ± 0.3	411 ± 8.5	19.7 ± 0.3	12.14
	2	7–24	18.10	1327 ± 9.5	29.59 ± 0.2	411 ± 7.5	24.08 ± 0.2	18.86
	3	24–36	16.56	1327 ± 8.5	32.12 ± 0.2	411 ± 5.2	23.35 ± 0.1	17.27
	4	36–48	16.64	1327 ± 5.3	38.36 ± 0.0	411 ± 6.5	26.76 ± 0.0	21.39

The MFCs also behave differently in terms of ${NH}_{4}-N$ removal. The removal percentages range from 19.7 ± 0.3 to 61.50 ± 0.0%). At an influent concentration of 411 ± 6 mg L^−1^ at 90% leachate and 10% v/v inoculation, MFC C has the maximum NH_4_-N removal efficiency of 61.50%. This is due to the microbial community’s optimally suited habitat for the denitrification process, a microbially aided process that reduces nitrate (NO_3_) and eventually creates molecular nitrogen (N_2_) via a sequence of intermediate gaseous nitrogen oxide products. Denitrification can remove both nitrate and ammonium nitrogen from wastewater. Additionally, as the bacterial population grows, so does the amount of organic matter and NH_4_-N accessible for removal, resulting in a higher proportion of COD and ${NH}_{4}-N$ removal (Hassan et al. 2019). Furthermore, when the bacterial population grows, they can produce more electrons during oxidation, resulting in higher Coulombic efficiency (CE), increased power output, and biofilm growth on the anode surface. The biofilm can operate as a barrier to prevent inhibitory substances from being transported and stimulate the growth of electroactive bacteria, resulting in greater COD and ammonia removal efficiency. Throughout the experiment, the levels of nitrogen (*TN*, ${NH}_{4}^{+}-N$, ${NO}_{3}^{-}-N,$ *and* ${NO}_{2}^{-}-N$) were measured and analysed. The removal effectiveness increased during the first 3 cycles of the experiment but then decreased later when the leachate concentration was changed after each cycle (Table 2), primarily mitigated as a result of the input of ${NH}_{4}^{+}-N$. According to Puig et al. (2011), the cationic exchange membrane (CEM) crossover to the cathodic chamber may be responsible for the removal of the majority of the ${NH}_{4}^{+}$*-N* in anaerobic conditions. The effective transport of electrons from the anode to the cathode electrode is made possible by the network of microorganisms provided by a mature biofilm. Metabolic pathways and interactions between microbes inside a biofilm are optimised during its development, leading to enhanced electron release and capture (Sehar and Naz 2016).

### Inhibition of electricity generation in continuous batch mode MFCs

**Fig. 3:**
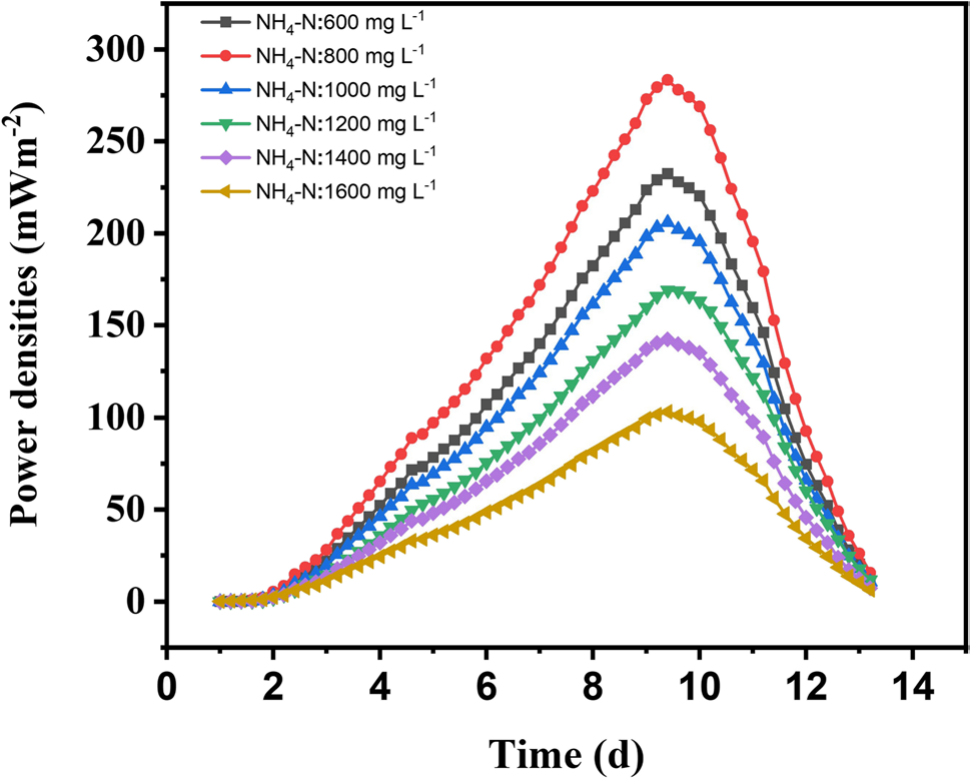
The average ammonia infractions with their respective power densities

Double-chamber MFCs were operated continuously at a constant operating condition to investigate the inhibitory effect of high ammonia nitrogen concentrations. On the other hand, with the addition of NH_4_Cl, to change TAN concentration from 600 to 1600 mgL^−1^, the reactors exhibited a power density (Fig. 3) which was greater than the power densities obtained when there was no ammonia addition. The ${NH}_{4}Cl$ in the influent solution was adjusted, and results were obtained (Fig. 3), which shows the average power densities of respective MFCs in different NH_3_ infractions. The experiment results show that as the concentration of ammonia nitrogen increases, the electricity generation in the MFC decreases. This conforms with the previous studies of Chamem et al. (2020), Ergettie and Dagbasi (2021), Gu et al. (2021), Gutierrez et al. (2016), Hiegemann et al. (2018), Ishaq et al. (2023), Khalil et al. (2017), Ren et al. (2021), Tice and Kim (2014), and Wang et al. (2021). Bacteria adhering to the anode cultured in continuous MFCs may have developed a tolerance to the high nitrogen concentration at which they are grown. The findings of this research provide strong evidence for the concept’s validity, showing that anode-attached bacteria may adjust so that their electricity-generating mechanism continues to function in any continuous influent with TAN concentrations up to 800 mg L^−1^.

**Fig. 4:**
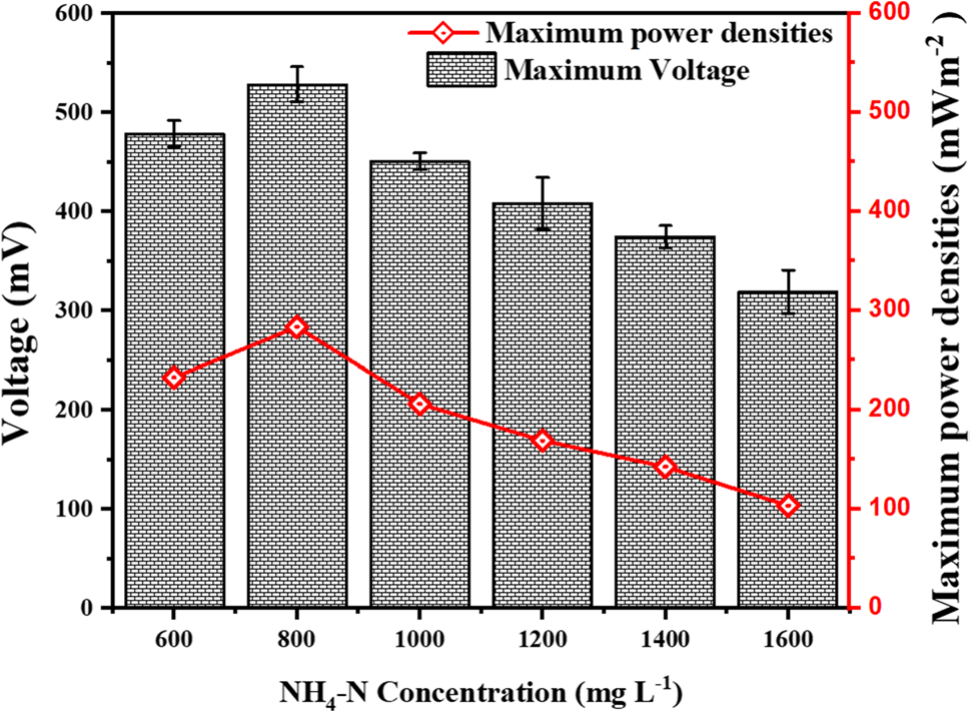
The maximum power densities of the respective MFCs at different ${{\text{NH}}}_{4}-{\text{N}}$ concentrations

Figure 4 shows the maximum power densities of the continuous double-chamber MFCs at ${{\text{NH}}}_{4}-{\text{N}}$ influent concentrations. Increase 600 mg L^−1^, 800 mg L^−1^, 1000 mg L^−1^, 1200 mg L^−1^, 1400 mg L^−1^ to 1600 mg L^−1^ results in an increase in steady-state power density 232.38 mWm^−2^ CE (17.12%), 283.38 mWm^−2^ CE (24.98%), 205.97 mWm^−2^ CE (18.87%), 168.98 mWm^−2^ CE (15.62%), 142.39 mWm^−2^ CE (14.62%), and 103.14 mWm^−2^ CE (12.03%) with corresponding voltages 478.67 mV, 528.59 mV, 450.66 mV, 408.18 mV, 374.70 mV, and 318.89 mV, respectively. The highest power density recorded is 283.3846 mWm^−2^ CE (24.98%) at 800 mg L^−1^, with the corresponding voltage 528.5993 mV (Fig. 4). Upon applying higher concentrations ranging from 1000 to 1600 mgL^−1^, pH (6.8), and COD (1332.5 mg L^−1^), the power density rapidly decreases to a minimum 103.14 mWm^−2^ CE (12.03%) at 1600 mgL^−1^. This is likely due to ammonia’s inhibitory effect on the microorganisms responsible for generating the electricity in the MFC. It can be concluded that the reactor’s power density (${NH}_{4}-N$, 800 mg L^−1^; COD, 1322.5; pH, 6.8) was greater than all the reactors. Moreover, the power density curve is a better parameter for predicting ammonia inhibition than continual monitoring of electric current. Thus, the power density results showed that the performance of the MFCs under high ammonia conditions could be decreased by increasing the concentration of the ${NH}_{4}-N$ and reducing the frequent feed of the reactors. The highest CE is observed at an ${NH}_{4}-N$ influent concentration of 800 mg L^−1^, with a value of 24.98%. As the ${NH}_{4}-N$ influent concentration deviates from this optimum, the CE values tend to decrease, reaching their lowest value of 12.03% at 1600 mg L^−1^. The higher CE in MFC (${NH}_{4}-N$, 800 mg L^−1^; COD, 1322.5) obtained was a result of the system efficiently converting the available organic matter in the leachate into electricity during each batch cycle, and the microorganisms in the MFC efficiently transferring electrons from the organic compounds to the anode, leading to a higher proportion of these electrons being converted into usable electrical energy (Cecconet et al. 2018) Furthermore, high concentrations of ${NH}_{4}-N$ may lead to increased competition among microorganisms for available electron acceptors, limiting the efficiency of electron transfer and thus reducing CE. Additionally, at higher ${NH}_{4}-N$ concentrations, there may be an accumulation of toxic byproducts or inhibitory substances that can negatively affect the metabolic activities of the microorganisms, further reducing CE.

Ammonia can act as a substrate for some microorganisms, but at high concentrations, it can become toxic and inhibit their growth and metabolism (Kim et al. 2007, 2011; Lee et al. 2021; Tice and Kim 2014). Low anolyte pH favours ionised ammonium (${NH}_{4}^{+}$) over unionised ammonia (${NH}_{3}$), which has a toxic effect on exoelectrogenic bacteria due to the high concentration of leachate and the frequent feed of the MFC (Ergettie and Dagbasi 2021). Increasing the amount of ${NH}_{3}$ in the system speeds up the breakdown of the substrate, leading to increased power output. In contrast, anammox bacteria experience self-inhibition and decreased power production when exposed to ${NH}_{3}$ concentrations over a particular limit (Chen et al. 2014; Jiang et al. 2014). Beyond a certain limit, an inverse link was seen between ${NH}_{3}$ concentration and power generation, consistent with previous research (Albarracin-Arias et al. 2021; Chang et al. 2018).

**Fig. 5:**
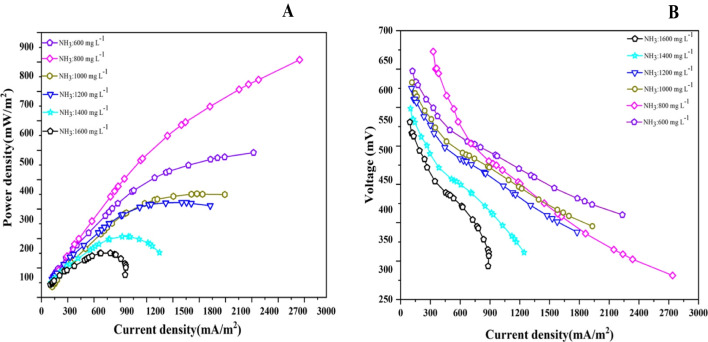
**A**–**B** Graphs of power density and voltage as a function of current density in batch MFC with varying ammonia concentrations

The polarisation curve charts illustrate variations in voltage and current density produced by the MFC in response to changes in ammonium nitrogen content, ranging from 600 to 1600 mgL^–1^. These curves visually depict the MFC’s response to diverse ammonium nitrogen concentrations. Maximum power output, recorded polarisation graphs, and normalised power density based on the anode surface area were assessed to evaluate the MFC reactor’s performance. In Fig. 5A, the highest power and current densities recorded were 855.31 mWm^–2^ and 2945.25 mA/m^2^ at an ammonium nitrogen concentration of 800 mg L^–1^. Additionally, power densities at ammonium nitrogen concentrations of 600, 1000, 1400, and 1600 mg L^–1^ were 491.96, 370.19, 322.40, 206.88, and 150.64 mWm^–2^, respectively. These results highlight the significance of ammonium nitrogen concentration in electrical power generation. Current density, measured at each ammonium nitrogen concentration, increases proportionally with voltage until reaching its maximum feasible value, as shown in Fig. 5B. The MFC operated with an ammonium nitrogen level of 800 mg L^–1^ demonstrated the highest power density, indicating optimal energy generation. Power densities declined beyond 800 mgL^–1^, indicating decreased MFC performance. This pattern underscores that elevated ammonium nitrogen concentrations do not necessarily correlate with increased MFC power densities (Ishaq et al. 2023). Biological processes contributed to a steady rise in cell voltage until reaching a maximal value (steady state; Fig. 5B), characteristic of a well-established microbial colony in the anode region under ideal conditions. The highest electricity generation from leachate wastewater can be influenced by microbiological activity and substrate quality, considering factors such as porosity, substance, and roughness during adsorption.

### Organics removals

#### Ammonia nitrogen removal

The initial concentrations of ammonium nitrogen (*NH*_*4*_^+^*-N*) and nitrite nitrogen (NO_2_^–^-N) were set according to a stoichiometric ratio of 1:1.132. This ratio is a crucial parameter in the anammox process for maintaining a specific *NO*_*2*_^–^*-N* to *NH*_*4*_^+^*-N* ratio, with an ideal influent *NO*_*2*_^–^*-N* to *NH*_*4*_^+^*-N* ratio within the theoretical range of 1.0 to 1.5, as suggested by previous studies (Wang et al. 2017). This ratio represents the oxygen required to fully oxidise the organic matter in the leachate. In anammox biofilm-enriched MFCs, ammonia, nitrite, and nitrate dynamics are complex, involving the sequential oxidation of ammonium to nitrite and then to nitrogen gas (Lu et al. 2020; Lu et al. 2021). This process results in the simultaneous removal of ammonium and nitrite from the leachate. However, nitrate, though typically found in lower concentrations, can impact the anammox process by competing with nitrite for the electrons generated by anammox bacteria (Gu et al. 2021). Some bacteria in the anammox biofilm can reduce nitrate to nitrite, diverting electrons and reducing the efficiency of the anammox process, compromising nitrogen removal.

**Fig. 6:**
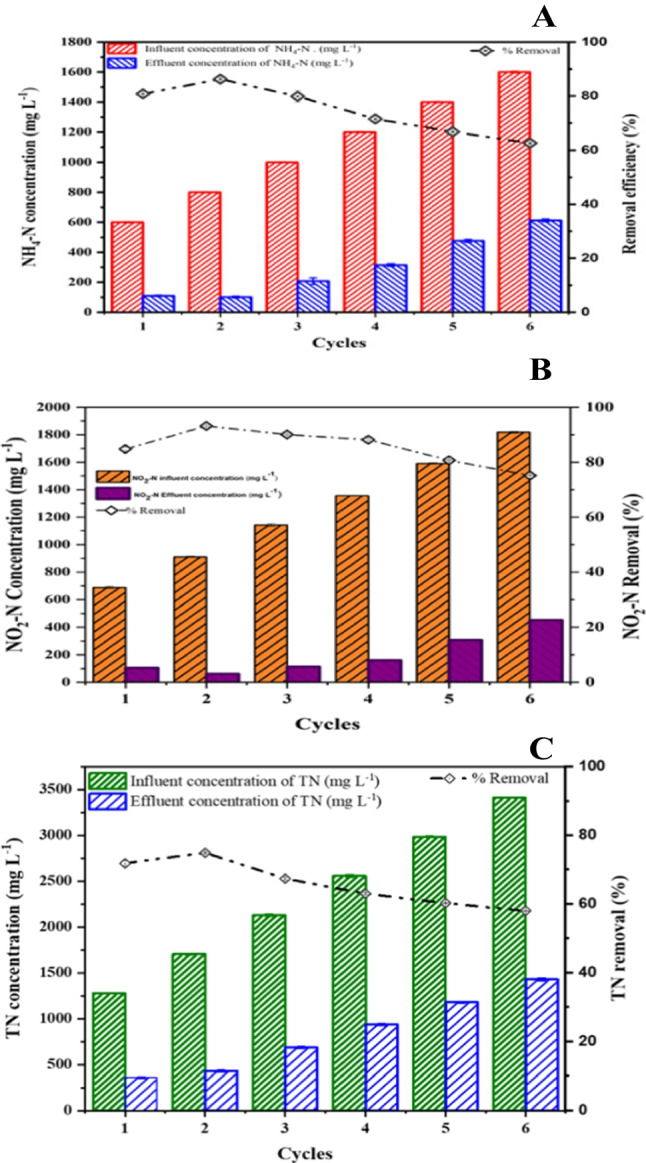
**A**–**C** The profiles of ${({\text{NH}}}_{4}^{+}$-N, ${{\text{NO}}}_{2}^{-}$-N, and TN) in both the influent and the effluent and how effectively they may be removed utilising landfill leachate as substrate

Figure 6A reveals that the percentage of *NH*_*4*_*-N* removed increased with the number of cycles. At an initial *NH*_*4*_*-N* concentration of 600 ± 5–800 ± 4.5 mg L^−1^, the removal efficacy ranged from 81.14 ± 0.6 to 86.40 ± 0.7% (cycles 1–2). This increased with further ammonium content (cycles 3–6), diverging the power output and ammonium removal trend. The highest *NO*_*2*_^–^*-N* removal efficiencies of 84.79 ± 0.5%, 93.1 ± 0.3%, and 90.04 ± 0.2 occurred at cycles 1, 2, and 3, gradually decreasing to 75.18 ± 0.1% at cycle 6. This suggests that *NH*_*4*_*-N* posed a toxic effect on anodic microorganisms, leading to a decreased nitrogen removal rate due to the inhibition effect and reduced removal efficiencies. Some microorganisms may be more susceptible to high *NH*_*4*_*-N* than others, leading to imbalances in the microbial community structure. High *NH*_*4*_*-N* concentrations can disrupt the integrity and permeability of microbial cell membranes. *NH*_*4*_*-N* can penetrate the cells and damage the lipid bilayer, affecting the transport of nutrients, ions, and metabolic intermediates across the cell membrane, impairing cellular processes, and compromising the viability and activity of anodic microorganisms (Gu et al. 2021).

The total nitrogen (TN) removal efficiencies were 71.21 ± 0–75.24 ± 0.2 in cycles 1–2, gradually decreasing to 67.04 ± 0.3–63.05 ± 0.3 in cycles 3–4, with the lowest removal of 58.06 ± 0.10 in cycle 4. The decrease in TN removal efficiency might be due to the common phenomenon of *NO*_*3*_^–^*-N* production in the anammox process. This further explains that in the anammox system, ammonia nitrogen, nitrite removal, and nitrate production occur from influent to effluent concentrations. Removing nitrogen may involve participation from a distinct conceivable pathway, as depicted in Fig. 6. The presence of anammox in the MFC system was confirmed by simultaneously removing *NH*_*4*_^+^*-N, NO*_*2*_^–^*-*N in an oxygen-depleted environment (Fig. 6). Anammox accounted for 7.4% of all anodic bacteria detected. This process is carried out by anoxic anammox bacteria, which results in the rapid conversion of *NH*_*4*_^+^*-N, NO*_*2*_^–^*-N*, into nitrogen gas (N_2_). The constant decrease in NO_2_^–^-N concentration throughout the experiment indicates that nitrification was hampered during anoxic conditions. This experiment established beyond a shadow of a doubt that anammox bacteria were responsible for the oxidation of *NH*_*4*_^+^*-N*. This observation validates the findings of Hassan et al. (2018), Pierangeli et al. (2021), Wang et al. (2019), and Wu et al. (2020), revealing that NH_4_^+^-N may cross through the membrane and reach the cathode chamber.

As a result, the removal efficiency of ammonia in MFCs occurred; the anode chamber’s microorganisms oxidise the organic matter in the leachate and release electrons and protons. The electrons are transferred to the cathode chamber through an external circuit, where they react with oxygen and protons to produce water. The ammonia in the leachate will diffuse across the membrane to the cathode chamber and react with the oxygen to form nitrate, a less toxic form of nitrogen. This process is known as nitrification and requires the presence of nitrifying bacteria, which can oxidise ammonia to nitrate. It has long been believed that two different types of AOMs complete nitrification under aerobic conditions; AOB or AOA first use oxygen to oxidise ammonia to nitrite, which is then further oxidised to nitrate by NOB (Wei et al. 2011).

The microbial processes driving pollutant removal in microbial fuel cells (MFCs), particularly in the context of ammonia nitrogen impact, involve key players and interactions. Anammox bacteria, constituting 7.4% of anodic bacteria, play a vital role in sequentially oxidising ammonium (*NH*_*4*_^+^*-N*) to nitrite and then to nitrogen gas (N_2_). Concurrently, various microbial groups participate in the oxidation of ammonium to nitrite and the potential reduction of nitrate to nitrite. This competition for electrons among microorganisms, as highlighted by Gu et al. (2021), significantly influences the efficiency of the anammox process, emphasising the need for a detailed understanding of specific microbial contributions to nitrite and nitrate dynamics. Total nitrogen (TN) removal involves microbial pathways where ammonium and nitrite are transformed into nitrogen gas and nitrate, respectively. The observed decrease in TN removal efficiency, attributed to nitrate production, underscores the importance of delineating the specific microbial communities responsible for these transformations. Furthermore, the nitrification process is facilitated by ammonia-oxidising microorganisms (AOB or AOA), which oxidise ammonia to nitrite. Nitrite-oxidising bacteria (NOB) subsequently oxidise nitrite to nitrate, as suggested by Wei et al. (2011). These nitrifying bacteria contribute to the overall nitrogen removal efficiency in MFCs, converting ammonia into a less toxic form (nitrate).

Hence, a comprehensive understanding of microbial processes in MFCs necessitates recognising the crucial roles of anammox bacteria, various microbial groups in nitrite and nitrate dynamics, and nitrifying bacteria in pollutant transformation. This insight into microbial mechanisms enriches our comprehension of pollutant removal efficiency in MFCs, as evidenced by the studies of Lu et al. (2020; 2021), Gu et al. (2021), and Wei et al. (2011). Further research could provide a more detailed exploration of these microbial contributions, enhancing the overall understanding of ammonia nitrogen’s impact on pollutant removal in MFCs.

### Bacterial community structure of MFCs

**Fig. 7:**
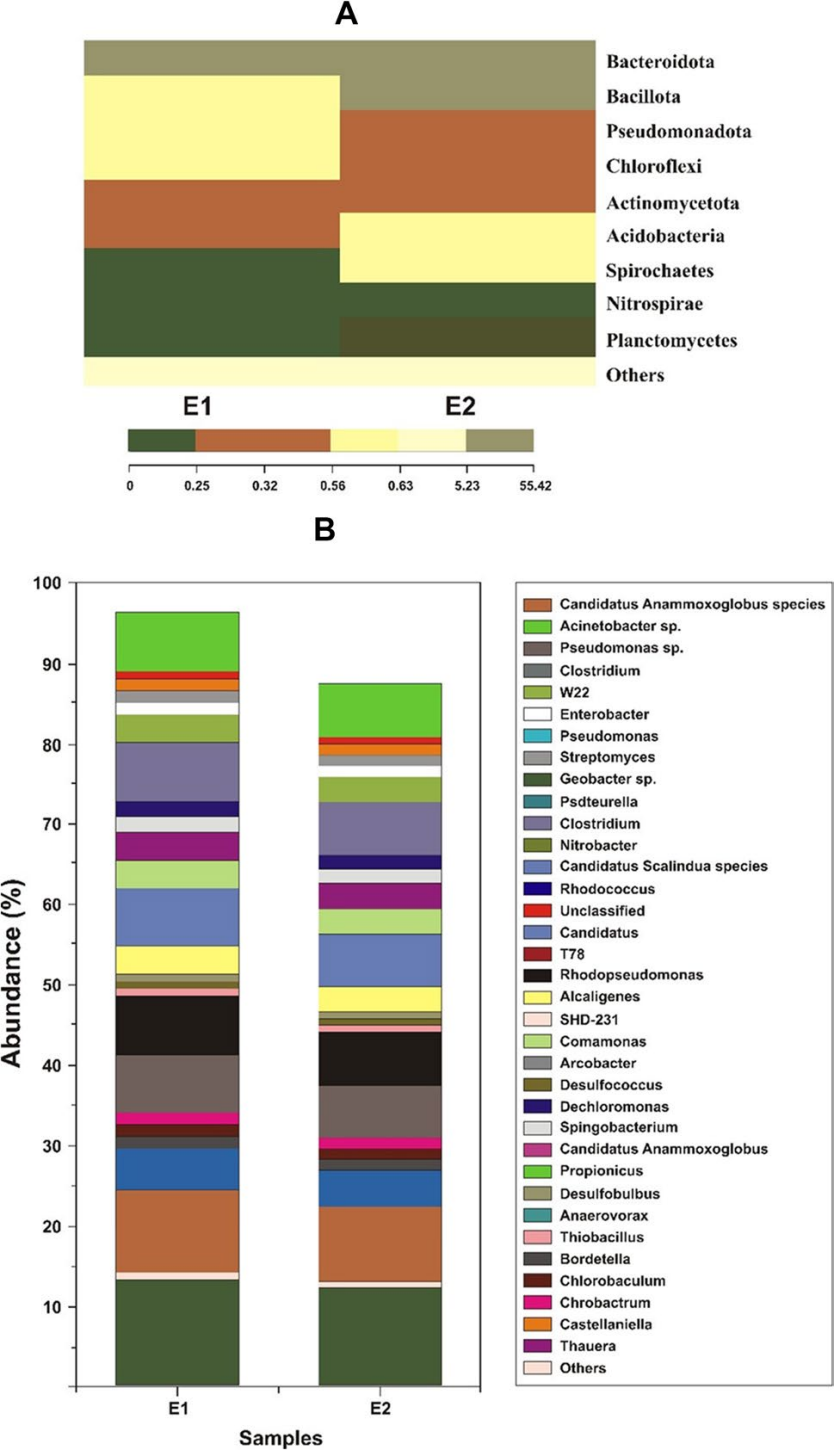
Bacterial community species sample in **A** and **B**

These findings are also illustrated in Fig. 7, which shows, via a network graph, the distribution of numerous species across the various samples. The density of each species is reflected in the size of the nodes that represent them, with smaller nodes representing lower densities. Species with abundances greater than 2% or rankings in the top 100 were specifically researched and analysed.

A comparison was made between the seven MFCs regarding the richness and diversity of the bacterial communities found on the anode electrode surfaces. It was determined that a total of 62,831 reads of high quality and 2108 operational taxonomic units (OTUs) were collected. Higher ACE and Chao1 index values indicate richer communities. According to Huang et al. (2018), the findings showed that MFCs with a higher richness were more advantageous for microbial enrichment. In addition, the Shannon index was 5.12, and the Simpson index was 0.15, demonstrating that the microbial community possessed a significant variety. Less variety is present when the Shannon and Simpson indexes are both low. One possible explanation for the proliferation of microorganisms in MFCs is the agitation caused by the cathode. These changes caused a rise in the population of anodic bacteria. On the anodes of all MFCs, the eight most common phyla were Proteobacteria, Nitrospirae, Peru bacteria, Chloroflexi, Acidobacteria, Planctomycetes, Actinomycetota, and Bacillota, making up more than 85% of the total microbial population. More than half of all microbes might be classified into these phyla. It is important to consider the potential that some of the bacteria on the list belong to more than one phylum because the classification of bacteria can be complex and is determined by a range of criteria. Electrochemically active bacteria are divided into four groups: Proteobacteria, Actinobacteria, Actinomycetes, and Firmicutes. These bacteria can perform electron transfer in two different ways. In this study, electrogenic bacteria comprised more than 14% of the categorised biofilm species. These electrogenic microorganisms were most likely accountable for electricity production in the MFCs.

In addition, ammonia-oxidising bacteria, nitrite-oxidising bacteria (such as *Nitrospira*), and denitrifying bacteria (including *Candidatus Scalindua* species, *Geobacter* sp, *Rhodopseudomonas*, *Comamonas*, and *Thauera*) were shown to be present in the anodic MFC systems (Tao et al. 2022). About 7.4% of the anodic species were accounted for by the relative abundance of nitrite-oxidising bacteria, most of which belonged to the genus *Planctomyces.* It is possible that these bacteria played a substantial part in the anammox oxidation process used in this investigation to reduce the amount of nitrogen. On the other hand, the anammox bacteria did not demonstrate very high removal efficiencies, which aligns with earlier research findings.

Anammox bacteria (6%), denitrifying bacteria (7.4%), and electrogenic bacteria (18.2%) were abundant in the anode’s microbial community, according to sequencing analysis.

## Conclusions

The MFCs were built and fed with landfill leachate with changing ammonia contents. This paper describes a novel method for optimising ${NH}_{4}-N$ at constant COD, pH, and ambient temperature for improved MFC performance. At an ${NH}_{4}-N$ of 800 mgL^−1^ and a COD content of 1322.5 mg L^−1^, the study produced a considerable power density of 234 mWm^−3^. The high ${NH}_{4}-N$ dosage improved overall system performance, but the inhibitory effect became more pronounced beyond a certain point. Nitrogen removal occurred using distinct removal pathways (66.0 3.3% ${NH}_{4}^{+}$*-N* and 86.0 0.1% ${NO}_{2}^{-}$*-N*). Sequencing analysis revealed that the anode hosted a diverse microbial community, with a high abundance of anammox (6%), denitrifying (7.4%), and electrogenic (18.2%) bacteria. Although pilot-scale experiments have shown promise, several limitations must be overcome before this technology can be widely implemented for leachate treatment and electricity generation.

## Recommendations

1. Further investigations should prioritise the identification of the most favourable ${NH}_{4}-N$ dose that achieves a harmonious equilibrium between improved performance of microbial fuel cells (MFCs) and the reported inhibitory effects that become apparent beyond a specific threshold. The optimisation of power generation in microbial fuel cells (MFCs) is of utmost importance to ensure a reliable and environmentally friendly energy production.
2. Further investigation might be conducted to optimise the conditions that promote specific routes for nitrogen removal, considering the separate removal processes for ${NH}_{4}^{+}$ and ${NO}_{2}^{-}-N$. This comprehension has the potential to enhance the effectiveness of nitrogen removal and the overall performance of MFCs.
3. Further research on the microbial community dynamics, especially the roles of anammox, denitrifying, and electrogenic bacteria, is recommended. Understanding how changes in NH_4_-N concentrations affect the abundance and activity of these microbial groups can provide insights into MFC performance.
4. Pilot-scale experiments have shown promise, but addressing and overcoming limitations for wider implementation is crucial. Investigate and resolve challenges related to scalability, robustness, and long-term stability for practical applications of MFC technology in leachate treatment and electricity generation.

## Data Availability

The manuscripts’ data is contained in the text.

## References

[CR1] Albarracin-Arias JA, Yu CP, Maeda T, Valdivieso Quintero W, Sanchez-Torres V (2021) Microbial community dynamics and electricity generation in MFCs inoculated with POME sludges and pure electrogenic culture. Int J Hydrog Energy 46(74):36903–36916. 10.1016/j.ijhydene.2021.08.218

[CR2] Association, A. P. H (2005) Standard methods for the examinations of waters and waste waters, 21st edn. APHA AWWA-WEF, Washington, DC

[CR3] Bakhshian S, Kariminia HR, Roshandel R (2011) Bioelectricity generation enhancement in a dual chamber microbial fuel cell under cathodic enzyme catalyzed dye decolorization. Bioresour Technol 102(12):6761–6765. 10.1016/j.biortech.2011.03.060 21511458 10.1016/j.biortech.2011.03.060

[CR4] Cecconet D, Molognoni D, Callegari A, Capodaglio AG (2018) Agro-food industry wastewater treatment with microbial fuel cells: energetic recovery issues. Int J Hydrog Energy 43(1):500–511. 10.1016/j.ijhydene.2017.07.231

[CR5] Chamem O, Fellner J, Zairi M (2020) Ammonia inhibition of waste degradation in landfills – a possible consequence of leachate recirculation in arid climates. Waste Manag Res 38(10):1078–1086. 10.1177/0734242X20920945 32356492 10.1177/0734242X20920945

[CR6] Chang H, Quan X, Zhong N, Zhang Z, Lu C, Li G, Cheng Z, Yang L (2018) High-efficiency nutrients reclamation from landfill leachate by microalgae Chlorella vulgaris in membrane photobioreactor for bio-lipid production. Biores Technol 266:374–381. 10.1016/j.biortech.2018.06.077 10.1016/j.biortech.2018.06.077 29982060

[CR7] Chen H, Zheng P, Zhang J, Xie Z, Ji J, Ghulam A (2014) Substrates and pathway of electricity generation in a nitrification-based microbial fuel cell. Bioresour Technol 161:208–214. 10.1016/j.biortech.2014.02.081 24704886 10.1016/j.biortech.2014.02.081

[CR8] Coppath Hamza HM, Duraisamy P, Periyasamy S, Pokkiladathu H, Muthuchamy M (2017) Simultaneous electricity generation and heavy metals reduction from distillery effluent by microbial fuel cell. Indian J Sci Technol 10(13):1–13. 10.17485/ijst/2017/v10i13/111203

[CR9] Damiano L, Jambeck JR, Ringelberg DB (2014) Municipal solid waste landfill leachate treatment and electricity production using microbial fuel cells. Appl Biochem Biotechnol 173(2):472–485. 10.1007/s12010-014-0854-x 24671566 10.1007/s12010-014-0854-x

[CR10] Deng Y, Zhu XX, Chen N, Feng C, Wang HHH, Kuang P, Hu W, Nguyen HTH, Kakarla R, Min B, Harshan AMKG, Lebron YAR, Moreira VR, Brasil YL, Silva AFR, Santos LVS de, Lange LC, Amaral MCS, Ibrahim RSB, … Asiri AM (2021) Synthesis of carbon nanotubes. Bioresour Technol 40(1):1–2010.1016/j.biortech.2016.02.006

[CR11] Ergettie AB, Dagbasi M (2021) Impact of wastewater concentration and feed frequency on ammonia inhibition in microbial fuel cells. Biofuels 12(6):655–661. 10.1080/17597269.2018.1519760

[CR12] Feng Q, Xu L, Xu Y, Liu C, Lu Y, Wang H, Wu T, Wang R, Chen Y, Cheng Y (2020) Treatment of aged landfill leachate by a self-sustained microbial fuel cell-microbial electrolysis cell system. Int J Electrochem Sci 15(1):1022–1033. 10.20964/2020.01.19

[CR13] Gokgoz M, Zhang W, Manage N, Mbengue M, Bolyard S, Chen J (2023) Survey on the current leachate treatments of public municipal solid waste landfills and the potential impact of per- and polyfluorinatedalkyl substances in the Eastern and Northwestern United States. J Air Waste Manag Assoc 73(8):638–648. 10.1080/10962247.2023.2235313 37431990 10.1080/10962247.2023.2235313

[CR14] Gu X, Huang Y, Hu Y, Gao J, Zhang M (2021) Inhibition of nitrite-oxidizing bacteria in automatic recycling PN/ANAMMOX under mainstream conditions. Bioresour Technol 342. 10.1016/j.biortech.2021.125935 10.1016/j.biortech.2021.125935 34571329

[CR15] Gutierrez J, Kwan TA, Zimmerman JB, Peccia J (2016) Ammonia inhibition in oleaginous microalgae. Algal Res 19:123–127. 10.1016/j.algal.2016.07.016

[CR16] Hassan H, Jin B, Donner E, Vasileiadis S, Saint C, Dai S (2018) Microbial community and bioelectrochemical activities in MFC for degrading phenol and producing electricity: microbial consortia could make differences. Chem Eng J 332:647–657. 10.1016/j.cej.2017.09.114

[CR17] Hassan MM, Haleem N, Baig MA, Jamal Y (2019) Phytoaccumulation of heavy metals from municipal solid waste leachate using different grasses under hydroponic condition. 3(2) (Article no.AJARR.46287):1–12. 10.1038/s41598-020-72800-2 10.1038/s41598-020-72800-2 PMC7519059 32978488

[CR18] Hiegemann H, Lübken M, Schulte P, Schmelz KG, Gredigk-Hoffmann S, Wichern M (2018) Inhibition of microbial fuel cell operation for municipal wastewater treatment by impact loads of free ammonia in bench- and 45 L-scale. Sci Total Environ 624:34–39. 10.1016/j.scitotenv.2017.12.072 29245036 10.1016/j.scitotenv.2017.12.072

[CR19] Huang L, Li X, Cai T, Huang M (2018) Electrochemical performance and community structure in three microbial fuel cells treating landfill leachate. Process Saf Environ Prot 113:378–387. 10.1016/j.psep.2017.11.008

[CR20] Hussein M, Yoneda K, Mohd-Zaki Z, Amir A, Othman N (2021) Heavy metals in leachate, impacted soils and natural soils of different landfills in Malaysia: an alarming threat. Chemosphere 267. 10.1016/j.chemosphere.2020.128874 10.1016/j.chemosphere.2020.128874 33199110

[CR21] Ichihashi O, Vishnivetskaya TA, Borole AP (2014) High-performance bioanode development for fermentable substrates via controlled electroactive biofilm growth. ChemElectroChem 1(11):1940–1947. 10.1002/celc.201402206

[CR22] Ishaq A, Said MIM, Azman SB, Abdulwahab MF, Jagun ZT (2023) Optimizing total ammonia–nitrogen concentration for enhanced microbial fuel cell performance in landfill leachate treatment: a bibliometric analysis and future directions. Environ Sci Pollut Res. 10.1007/s11356-023-28580 10.1007/s11356-023-28580-z PMC10404197 37454007

[CR23] Jagaba AH, Kutty SRM, Lawal IM, Abubakar S, Hassan I, Zubairu I, Umaru I, Abdurrasheed AS, Adam AA, Ghaleb AAS, Almahbashi NMY, Al-dhawi BNS, Noor A (2021) Sequencing batch reactor technology for landfill leachate treatment: a state-of-the-art review. In: Journal of Environmental Management. Academic Press, (282). 10.1016/j.jenvman.2021.111946 10.1016/j.jenvman.2021.111946 33486234

[CR24] Jiang A, Zhang T, Zhao Q-B, Li X, Chen S, Frear CS (2014) Evaluation of an integrated ammonia stripping, recovery, and biogas scrubbing system for use with anaerobically digested dairy manure. Biosyst Eng 119:117–126

[CR25] Jiang K, Zhou K, Yang Y (2019) Removal of ammonia from a smelting wastewater by cyclic stripping and acid adsorption: kinetics study. Environ Prog Sustain Energy 38(5):2–7. 10.1002/ep.13159

[CR26] Kartal B, de Almeida NM, Maalcke WJ, Op den Camp HJM, Jetten MSM, Keltjens JT (2013) How to make a living from anaerobic ammonium oxidation. FEMS Microbiol Rev 37(3):428–461 23210799 10.1111/1574-6976.12014

[CR27] Khalil CA, Ghanimeh S, Medawar Y (2017) Ammonia inhibition and recovery potential in anaerobic digesters: a review. Proceedings of the Air and Waste Management Association’s Annual Conference and Exhibition, AWMA, January 2021

[CR28] Kim D, Ryu HD, Kim MS, Kim J, Lee SI (2007) Enhancing struvite precipitation potential for ammonia nitrogen removal in municipal landfill leachate. J Hazard Mater 146(1–2):81–85. 10.1016/j.jhazmat.2006.11.054 17208368 10.1016/j.jhazmat.2006.11.054

[CR29] Kim HW, Nam JY, Shin HS (2011) Ammonia inhibition and microbial adaptation in continuous single-chamber microbial fuel cells. J Power Sources 196(15):6210–6213. 10.1016/j.jpowsour.2011.03.061

[CR30] Kumar SS, Kumar V, Gnaneswar Gude V, Malyan SK, Pugazhendhi A (2020) Alkalinity and salinity favor bioelectricity generation potential of Clostridium, Tetrathiobacter and Desulfovibrio consortium in microbial fuel cells (MFC) treating sulfate-laden wastewater. Bioresour Technol 306. 10.1016/j.biortech.2020.123110 10.1016/j.biortech.2020.123110 32172090

[CR31] Kurniawan TA, Lo W, Chan GY (2006) Physico-chemical treatments for removal of recalcitrant contaminants from landfill leachate. J Hazard Mater 126(28 November):80–100. 10.1016/j.jhazmat.2005.08.010 10.1016/j.jhazmat.2005.08.010 16314043

[CR32] Lee G, Kim K, Chung J, Han JI (2021) Electrochemical ammonia accumulation and recovery from ammonia-rich livestock wastewater. Chemosphere 270:128631. 10.1016/j.chemosphere.2020.128631 33172673 10.1016/j.chemosphere.2020.128631

[CR33] Li WW, Yu HQ, He Z (2014) Towards sustainable wastewater treatment by using microbial fuel cells-centered technologies. Energy Environ Sci 7(3):911–924. 10.1039/c3ee43106a

[CR34] Li S-L, Wang Y-J, Chen Y-C, Liu S-M, Yu C-P (2019) Chemical characteristics of electron shuttles affect extracellular electron transfer: shewanella decolorationis NTOU1 simultaneously exploiting acetate and mediators. Front Microbiol 10:399 30891020 10.3389/fmicb.2019.00399 PMC6411715

[CR35] Li M, Li YW, Yu XL, Guo JJ, Xiang L, Liu BL, Zhao HM, Xu MY, Feng NX, Yu PF, Cai QY, Mo CH (2020) Improved bio-electricity production in bio-electrochemical reactor for wastewater treatment using biomass carbon derived from sludge supported carbon felt anode. Sci Total Environ 726:138573. 10.1016/j.scitotenv.2020.138573 32311574 10.1016/j.scitotenv.2020.138573

[CR36] Logan BE (2010) Scaling up microbial fuel cells and other bioelectrochemical systems. Appl Microbiol Biotechnol 85(6):1665–1671. 10.1007/s00253-009-2378-9 20013119 10.1007/s00253-009-2378-9

[CR37] Lu H, Yu Y, Xi H, Zhou Y, Wang C (2020) A quick start method for microbial fuel cells. Chemosphere 259:127323. 10.1016/j.chemosphere.2020.127323 32593813 10.1016/j.chemosphere.2020.127323

[CR38] Lu N, Li L, Wang C, Wang Z, Wang Y, Yan Y, Qu J, Guan J (2021) Simultaneous enhancement of power generation and chlorophenol degradation in nonmodified microbial fuel cells using an electroactive biofilm carbon felt anode. Sci Total Environ 783. 10.1016/j.scitotenv.2021.147045 10.1016/j.scitotenv.2021.147045 34088112

[CR39] Nor MHM, Mubarak MFM, Elmi HSA, Ibrahim N, Wahab MFA, Ibrahim Z (2015) Bioelectricity generation in microbial fuel cell using natural microflora and isolated pure culture bacteria from anaerobic palm oil mill effluent sludge. Biores Technol 190:458–465 10.1016/j.biortech.2015.02.103 25799955

[CR40] Oliveira VB, Simões M, Melo LF, Pinto AMFR (2013) Overview on the developments of microbial fuel cells. In. Biochem Eng J 73:53–64. 10.1016/j.bej.2013.01.012

[CR41] Özkaya B, Cetinkaya AY, Cakmakci M, Karadaǧ D, Sahinkaya E (2013) Electricity generation from young landfill leachate in a microbial fuel cell with a new electrode material. Bioprocess Biosyst Eng 36(4):399–405. 10.1007/s00449-012-0796-z 22903571 10.1007/s00449-012-0796-z

[CR42] Pierangeli GMF, Ragio RA, Benassi RF, Gregoracci GB, Subtil EL (2021) Pollutant removal, electricity generation and microbial community in an electrochemical membrane bioreactor during co-treatment of sewage and landfill leachate. Journal of Environmental. Chem Eng 9(5). 10.1016/j.jece.2021.106205

[CR43] Puiga S, Serraa M, Comaa M, Marina Cabréa M, Dolors Balaguera JC (2011) Microbial fuel cell application in landfill leachate treatment. J Hazard Mater 18(5):763–767. 10.1016/j.jhazmat.2010.09.086 10.1016/j.jhazmat.2010.09.086 20970254

[CR44] Rahimnejad M, Adhami A, Darvari S, Zirepour A, Oh SE (2015) Microbial fuel cell as new technology for bioelectricity generation: a review. Alex Eng J 54(3):745–756. 10.1016/j.aej.2015.03.031

[CR45] Rahmani AR, Navidjouy N, Rahimnejad M, Alizadeh S, Samarghandi MR, Nematollahi D (2022) Effect of different concentrations of substrate in microbial fuel cells toward bioenergy recovery and simultaneous wastewater treatment. Environ Technol (united Kingdom) 43(1):1–9. 10.1080/09593330.2020.1772374 10.1080/09593330.2020.1772374 32431240

[CR46] Ren S, Wang Z, Jiang H, Qiu J, Li X, Zhang Q, Peng Y (2021) Stable nitritation of mature landfill leachate via in-situ selective inhibition by free nitrous acid. Biores Technol 340(June):125647. 10.1016/j.biortech.2021.125647 10.1016/j.biortech.2021.125647 34385123

[CR47] Renou S, Givaudan JG, Poulain S, Dirassouyan F, Moulin P (2008) Landfill leachate treatment: review and opportunity. J Hazard Mater 150(3):468–493. 10.1016/j.jhazmat.2007.09.077 17997033 10.1016/j.jhazmat.2007.09.077

[CR48] Sehar S, Naz I (2016) Role of the biofilms in wastewater treatment. In: Microbial Biofilms - Importance and Applications. InTech. 10.5772/63499

[CR49] Tao X, Hu X, Wen Z, Li J, Liu Y, Chen R (2022) Highly efficient Cr (VI) removal from industrial electroplating wastewater over Bi2S3 nanostructures prepared by dual sulfur-precursors: insights on the promotion effect of sulfate ions. J Hazard Mater 424:127423 10.1016/j.jhazmat.2021.127423 34649121

[CR50] Tatsi AA, Zouboulis AI (2002) A field investigation of the quantity and quality of leachate from a municipal solid waste landfill in a Mediterranean climate (Thessaloniki, Greece). Adv Environ Res 6(3):207–219

[CR51] Tice RC, Kim Y (2014) Influence of substrate concentration and feed frequency on ammonia inhibition in microbial fuel cells. J Power Sources 271:360–365. 10.1016/j.jpowsour.2014.08.016

[CR52] Virdis B, Freguia S, Rozendal RA, Rabaey K, Yuan Z, Keller J (2011) Microbial fuel cells. In Treatise on Water Science (4). 10.1016/B978-0-444-53199-5.00098-1

[CR53] Wang H, Wang Y, Lou Z, Zhu N, Yuan H (2017) The degradation processes of refractory substances in nanofiltration concentrated leachate using micro-ozonation. Waste Manag 69:274–280. 10.1016/j.wasman.2017.08.048 28886976 10.1016/j.wasman.2017.08.048

[CR54] Wang H, Zheng X, Yan Q, Zhang G, Kim JR (2021) Microbial community and metabolic responses to electrical field intensity for alleviation of ammonia inhibition in an integrated bioelectrochemical system (BES). Biores Technol 336(May):125332. 10.1016/j.biortech.2021.125332 10.1016/j.biortech.2021.125332 34090099

[CR55] Wang Z, Zhang L, Zhang F, Jiang H, Ren S, Wang W, Peng Y (2019) Enhanced nitrogen removal from nitrate-rich mature leachate via partial denitrification (PD)-anammox under real-time control. Bioresour Technol 289(05 June):121615. 10.1016/j.biortech.2019.121615 31227428 10.1016/j.biortech.2019.121615

[CR56] Wei J, Liang P, Huang X (2011) Recent progress in electrodes for microbial fuel cells. Bioresour Technol 102(20):9335–9344. 10.1016/j.biortech.2011.07.019 21855328 10.1016/j.biortech.2011.07.019

[CR57] Wijekoon P, Koliyabandara PA, Cooray AT, Lam SS, Athapattu BCL, Vithanage M (2022) Progress and prospects in mitigation of landfill leachate pollution: risk, pollution potential, treatment and challenges. J Hazard Mater 421(May 2021):126627. 10.1016/j.jhazmat.2021.126627 34343881 10.1016/j.jhazmat.2021.126627

[CR58] Wu Q, Jiao S, Ma M, Peng S (2020) Microbial fuel cell system: a promising technology for pollutant removal and environmental remediation. Environ Sci Pollut Res 27(7):6749–6764 10.1007/s11356-020-07745-0 31956948

[CR59] Yaashikaa PR, Kumar PS, Nhung TC, Hemavathy RV, Jawahar MJ, Neshaanthini JP, Rangasamy G (2022) A review on landfill system for municipal solid wastes: insight into leachate, gas emissions, environmental and economic analysis. Chemosphere 309. 10.1016/j.chemosphere.2022.136627 10.1016/j.chemosphere.2022.136627 36181852

[CR60] Zhang F, He Z (2013) A cooperative microbial fuel cell system for waste treatment and energy recovery. Environ Technol (united Kingdom) 34(13–14):1905–1913. 10.1080/09593330.2013.770540 10.1080/09593330.2013.770540 24350444

[CR61] Zhao W, Bi X, Bai M, Wang Y (2023) Research advances of ammonia oxidation microorganisms in wastewater: metabolic characteristics, microbial community, influencing factors and process applications. Bioprocess Biosyst Eng. 10.1007/s00449-023-02866-5 10.1007/s00449-023-02866-5 36988685

[CR62] Zhang G, Jiao Y, Lee DJ (2015) Transformation of dissolved organic matters in landfill leachatebioelectrochemical system. Bioresour Technol 191:350–354. 10.1016/j.biortech.2015.05.082 26037237 10.1016/j.biortech.2015.05.082

